# Real-World Effectiveness of Polymerized House-Dust-Mite Subcutaneous Immunotherapy up to 24 Months: Why Baseline Symptom Severity Shapes Measurement of the Combined Symptom–Medication Response

**DOI:** 10.3390/jcm15166383

**Published:** 2026-08-18

**Authors:** Margarita Rosa Acevedo Matos, Omar Francisco Sierra Salgado, Yenny Alexandra Mendez, Sandra del Pozo, Miguel Casanovas

**Affiliations:** 1 AlergoIPS, Pereira 660003, Risaralda, Colombiaomarfs123123@gmail.com (O.F.S.S.);; 2 Inmunotek S.L., 28805 Alcalá de Henares, Madrid, Spain; mcasanovas@inmunotek.com

**Keywords:** house dust mite, allergen immunotherapy, subcutaneous immunotherapy, allergic rhinoconjunctivitis, real-world evidence, symptom–medication score, quality of life, floor effect

## Abstract

**Background/Objectives**: Allergic rhinoconjunctivitis (AR) induced by house dust mites (HDMs) is a common condition that impairs quality of life, and allergen immunotherapy is the only treatment accepted to modify its natural course. However, real-world evidence on polymerized HDM subcutaneous immunotherapy (SCIT) remains limited. We evaluated the up to 24-month effectiveness of polymerized HDM SCIT and examined how baseline symptom burden affects the measured response. **Methods**: In a retrospective, single-arm, real-world cohort, 353 patients (mean age 15.9 (12.3) years) with HDM-induced AR with/without asthma (asthma subpopulation, *n* = 112) initiated polymerized SCIT (ALXOID^®^, Inmunotek S.L., Spain) with an extract of house dust mites (*Dermatophagoides pteronyssinus*, *D. farinae*, *Blomia tropicalis*). Patients were followed for up to 24 months; 139 contributed an observation at month 12 and 67 at month 24. The primary outcome was the Rhinoconjunctivitis Combined Symptom–Medication Score (RCSMS); secondary outcomes were a visual analog scale (VAS) and the ESPRINT-15 quality-of-life questionnaire. Effectiveness was estimated using mixed models for repeated measures. The pre-specified primary-analysis population was restricted to a baseline RCSMS ≥ 2 to avoid floor effects and regression to the mean at low baseline scores. **Results**: At month 24, the RCSMS decreased by 0.938 points (−35.3%) in the full cohort and by 48.3% in the baseline RCSMS ≥ 2 population; in the full cohort, the VAS and ESPRINT-15 improved by 50.6% and 69.7%, respectively (all *p* < 0.001; within-subject effect sizes were small for the full-cohort RCSMS and medium for all other outcomes). The shape of the improvement trajectory did not differ by age group, and all age groups exceeded the minimal clinically important difference; the age analysis was exploratory. Patients with baseline RCSMS < 2 showed continued improvement in VAS and ESPRINT-15 scores. In contrast, the RCSMS increased, a pattern consistent with a statistical floor effect, suggesting limited responsiveness of the score at low baseline values. **Conclusions**: Up to 24 months, polymerized HDM SCIT was associated with clinically meaningful improvements in symptoms, medication use, and quality of life, most clearly in patients with a baseline RCSMS ≥ 2. Baseline symptom severity should be considered when interpreting combined symptom–medication outcomes in allergen immunotherapy. The single-arm design precludes causal inference.

## 1. Introduction

Allergic rhinitis affects 10–40% of the population worldwide and is a leading cause of impaired quality of life and healthcare expenditure [1]. House dust mites are among the most important perennial indoor allergens [2], and in tropical and subtropical regions, *Blomia tropicalis* is a major additional sensitizing species [3,4]. Allergen immunotherapy is the only treatment able to modify the natural course of IgE-mediated disease, including house dust mite allergy [5,6,7], and polymerized (allergoid) extracts allow higher cumulative doses with fewer injections [8,9].

International task forces recommend the RCSMS as the primary endpoint for AIT studies because it integrates symptom burden with the rescue medication required to control it [10]. By design, the score is bounded below: a patient who begins with little symptom–medication burden has little room to improve on the scale. This leads to two statistical consequences: (i) a floor effect means genuine improvement cannot be numerically expressed near the lower bound; and (ii) regression to the mean (RTM) means patients observed at a transiently low value tend to drift upward on re-measurement, producing an apparent worsening that is a statistical artifact rather than treatment failure [11,12]. Both effects are expected to be greatest in patients with the lowest baseline scores.

To mitigate this, a minimum baseline RCSMS has been proposed for effectiveness analyses. In mite allergy, a phase 3 trial independently converged on a comparable severity threshold: its full-cohort primary analysis, diluted by a majority of mildly symptomatic patients, did not meet the endpoint, whereas a post hoc subgroup with a baseline nasal symptom score ≥ 2 did [13]. Because that threshold was defined on a nasal symptom score rather than on the combined symptom–medication score used here and was reached post hoc, and because our own ≥ 2 threshold had been pre-specified in the study protocol before that trial was published, we regard this convergence as independent external corroboration rather than the origin of our choice. Real-world cohorts, however, span the entire severity range, which is exactly what makes a bounded composite score difficult to interpret, yet real-world evidence is increasingly central to regulatory and clinical decision-making [14,15]. Consistent with this, a recent real-world grass-pollen cohort assessed with the CSMS found that roughly a third of AIT-interested patients fell below the ≥1.5 eligibility threshold, and the interpretation of the composite depended on the severity distribution—a measurement issue demonstrated for a different allergen that parallels the one addressed here [16].

In this 24-month real-world cohort of polymerized HDM subcutaneous AIT, we therefore set out to (i) quantify effectiveness across three complementary instruments; (ii) test whether baseline severity influences the interpretability of the RCSMS; and (iii) evaluate a pre-specified baseline ≥ 2 effectiveness analysis using the excluded group and an explicit RTM analysis to test, rather than assume, the rationale for the threshold.

## 2. Materials and Methods

### 2.1. Design and Setting

This study comprises a retrospective, single-arm, real-world cohort of 353 patients from a single allergy center (AlergoIPS, Pereira, Colombia), reported per STROBE and RECORD [17,18]. Patients who initiated treatment between June 2021 and May 2025 were included. Data came from a single fixed extraction of the clinical records. As treatment was initiated at different calendar times, follow-up duration varied across individuals. Missing later visits, therefore, reflect administrative right-censoring imposed by the fixed observation window, not dropout or treatment discontinuation. Visit-by-visit values for all instruments and populations are given in Supplementary Tables S1–S3, with the corresponding trajectories in Supplementary Figures S1 and S2 and the observed count per visit in Supplementary Figure S5.

### 2.2. Participants and Intervention

Adults, adolescents and children with HDM allergic rhinoconjunctivitis, with or without concomitant asthma, who initiated subcutaneous AIT were eligible. The product combined allergenic extracts of *Dermatophagoides pteronyssinus*, *Dermatophagoides farinae* and *Blomia tropicalis*, each individually polymerized with glutaraldehyde and subsequently combined (manufacturer: Inmunotek S.L., Madrid, Spain; marketed in Colombia as ALXOID^®^). It was supplied as a 10,000 TU/mL suspension for injection containing equal parts of the three individually polymerized extracts, adsorbed onto aluminum hydroxide [9,19].

Because the analysis is retrospective, eligibility was operationalized at the point of record extraction rather than applied prospectively. The eligibility criteria were IgE-mediated respiratory allergy due to sensitization to *Dermatophagoides pteronyssinus*, *D. farinae* and *Blomia tropicalis*; a positive skin-prick test; no clinically relevant sensitization to other aeroallergens requiring allergen immunotherapy; and patients who were considered clinically evaluable by the investigator. Patients with a history of previous aeroallergen immunotherapy were excluded.

### 2.3. Outcomes

Primary: RCSMS (theoretical range 0–6) [10]. Secondary: ESPRINT-15 quality-of-life questionnaire (scale 0–6) [20,21], a VAS of global severity (scale 0–10), and, in asthmatics, an asthma composite symptom–medication score (ACSMS) [22]. MCID was pre-specified as a ≥20% relative RCSMS reduction, ≥2 VAS points, and ≥0.5 ESPRINT-15 points [23,24].

### 2.4. Population with a Baseline RCSMS ≥ 2 and Statistical Analysis

A pre-specified effectiveness population was defined by baseline RCSMS ≥ 2. Trajectories of the full cohort and of the population with a baseline RCSMS ≥ 2 were estimated by mixed models for repeated measures (MMRMs) with a first-order autoregressive [AR(1)] covariance structure and fitted by restricted maximum likelihood (REML), yielding estimated marginal means (EMMs). Baseline (V1) entered the model as a covariate and not as a response, since no treatment exposure occurred at the first visit; the response vector comprised the post-baseline visits [25,26]. Under the administrative right-censoring mechanism, missingness was assumed to be consistent with a missing-at-random (MAR) mechanism, under which the likelihood-based MMRM is valid without imputation [27,28]. Robustness was checked with complete-case, last-observation (LOCF), and tipping-point/delta-MI analyses [29]. Within-subject (Cohen’s d_z) and baseline-standardized (Glass’s Δ) effect sizes were reported. To test the threshold, the last available RCSMS was regressed on baseline RCSMS, and the break-even baseline was estimated [11,12]. Age-group × time interactions were tested in the full cohort and ≥2 populations; models compared in likelihood-ratio tests were fitted by maximum likelihood rather than REML, as REML log-likelihoods are not comparable across models differing in their fixed-effects structure. This study is single-arm and uncontrolled; all statements are associative.

## 3. Results

### 3.1. Study Population

Three hundred fifty-three patients were analyzed (Table 1); 259 (73.4%) had a baseline RCSMS ≥ 2 and 94 (26.6%) < 2. Groups were comparable in sex and age distribution, and by definition, they differed in baseline RCSMS. Additional aeroallergen sensitization was recorded in 114 patients (32.3%), with similar proportions in the RCSMS ≥ 2 and <2 groups (32.8% and 30.9%, respectively; Table 1). These included animal epithelia (including cat and dog), cockroach and grass pollen (Supplementary Table S8). Concomitant asthma and atopic dermatitis were recorded in 112 (31.7%) and 48 (13.6%) patients, respectively. As expected, the populations differed in baseline RCSMS; baseline VAS and ESPRINT-15 scores were also higher in the RCSMS ≥ 2 population (Table 1).

Baseline sex and age distribution, RCSMS and VAS were similar between patients with and without an available month-24 RCSMS assessment (Supplementary Table S11; all *p* > 0.05).

### 3.2. RCSMS Effectiveness Depends on Baseline Severity

**Figure 1:**
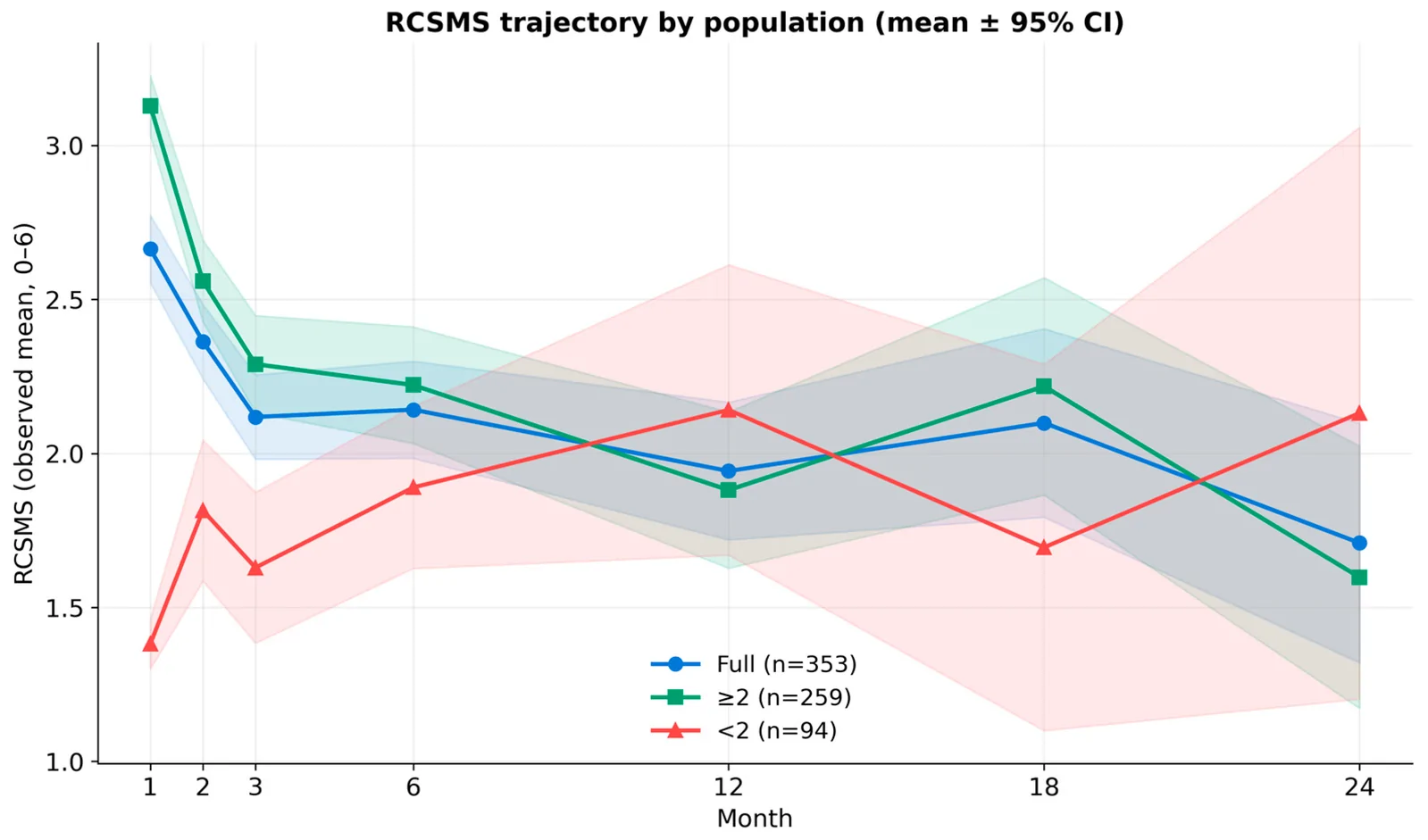
RCSMS trajectory (observed mean ± 95% CI) at the scheduled visits for the full cohort, the baseline ≥ 2 population, and the baseline < 2 group. The ≥2 population falls steeply, whereas the <2 group rises above its own baseline.

Immunotherapy was associated with improvement across the entire cohort on all three instruments; however, the RCSMS most clearly reflected this improvement when the analysis was restricted to patients whose baseline score was ≥2. In the full cohort, the RCSMS decreased 35.3% at month 24 (MCID first reached at month 12; d_z −0.33), whereas the secondary instruments improved far more (ESPRINT-15 −69.7%; VAS −50.6%), suggesting that the RCSMS, applied to the whole severity range, understates the response. Restricting to the pre-specified ≥2 population produced a larger, earlier and more stable RCSMS reduction of 48.3% (MCID sustained from month 3; d_z −0.62), concordant with ESPRINT-15 (−64.1%) and VAS (−51.6%) (Table 2; Figure 1). The contrast in timing makes the dilution concrete. Once the group with very low baseline RCSMS was excluded, the results demonstrated −20% MCID at month 3 rather than month 12, an earlier and more stable improvement that reflects restored measurability of the bounded endpoint rather than a faster clinical response. Of the 259 patients with a baseline RCSMS ≥ 2, 232 contributed at least one post-baseline RCSMS assessment and were included in the MMRM analysis; the remaining 27 had no post-baseline RCSMS observations because, in this retrospective study, data were extracted within a fixed observation window. The number of patients with available observations at each visit is reported in the corresponding tables and Supplementary Material; for RCSMS, 139 and 67 patients had observations at months 12 and 24, respectively.

### 3.3. The Excluded <2 Group: Discordance, Not Failure

**Figure 2:**
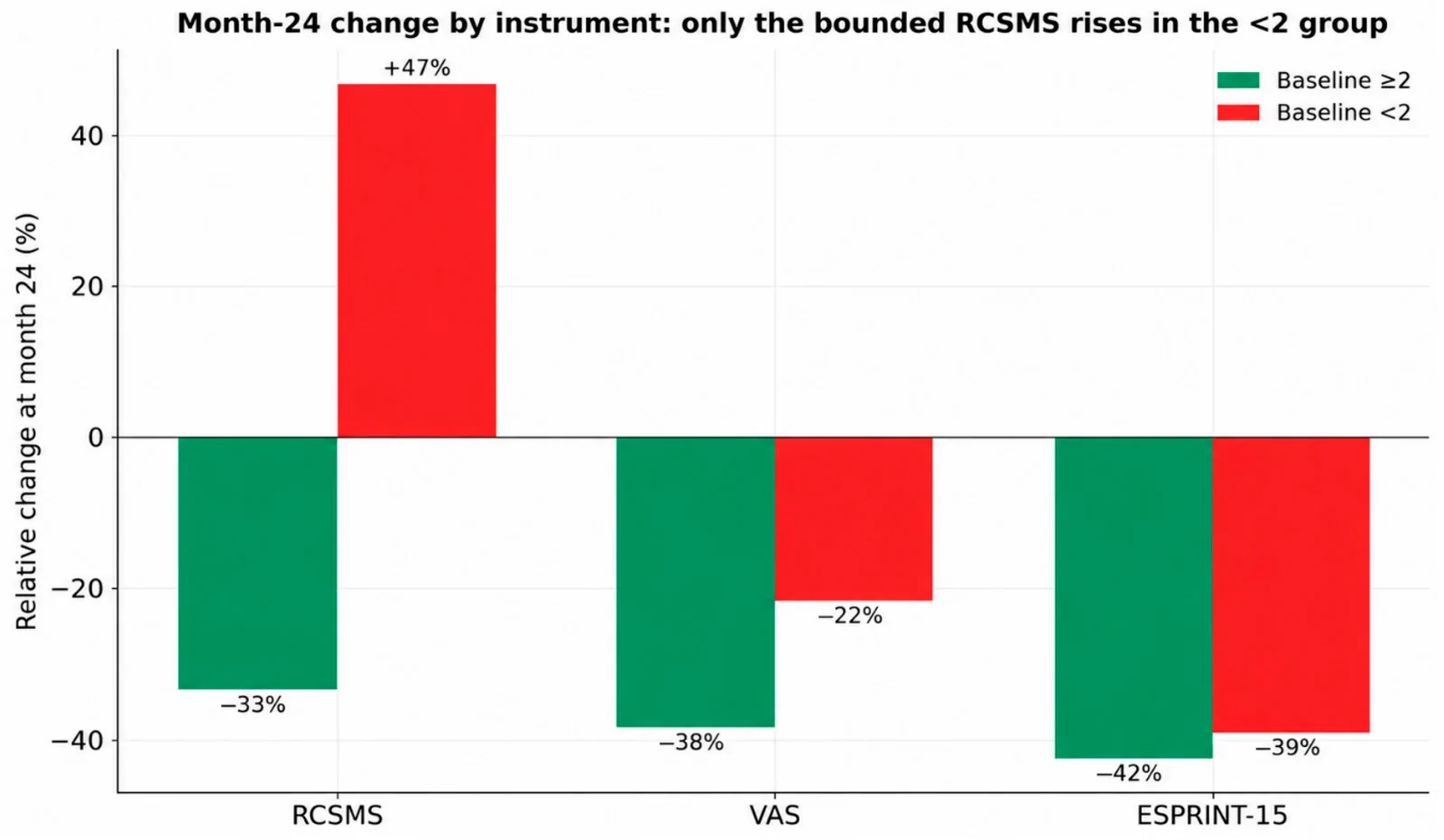
Change at month 24 by instrument in the ≥2 and <2 populations. Only the bounded RCSMS rises in the <2 group; quality-of-life (ESPRINT-15) and global severity (VAS) improve in both.

In the low-baseline population, the RCSMS increased by 64.2% at month 24, whereas ESPRINT-15 and VAS improved by 83.2% and 45.7%, respectively (Figure 2). A treatment-related deterioration would worsen all three instruments; instead, only the bounded RCSMS rose, while the two instruments not constrained by the same floor improved markedly. This dissociation is consistent with floor and regression-to-the-mean effects at low baseline values, indicating a measurement limitation rather than lack of clinical benefit. Analysis of the RCSMS components showed different patterns between populations (Supplementary Table S9). In the baseline RCSMS ≥ 2 population, both RSS and RMS generally decreased during follow-up. In the baseline RCSMS < 2 population, RSS remained broadly stable across visits, whereas RMS tended to increase over time, suggesting that the rise in the composite score may have been more closely related to the medication component than to worsening symptoms.

### 3.4. Regression to the Mean and the Break-Even Baseline

**Figure 3:**
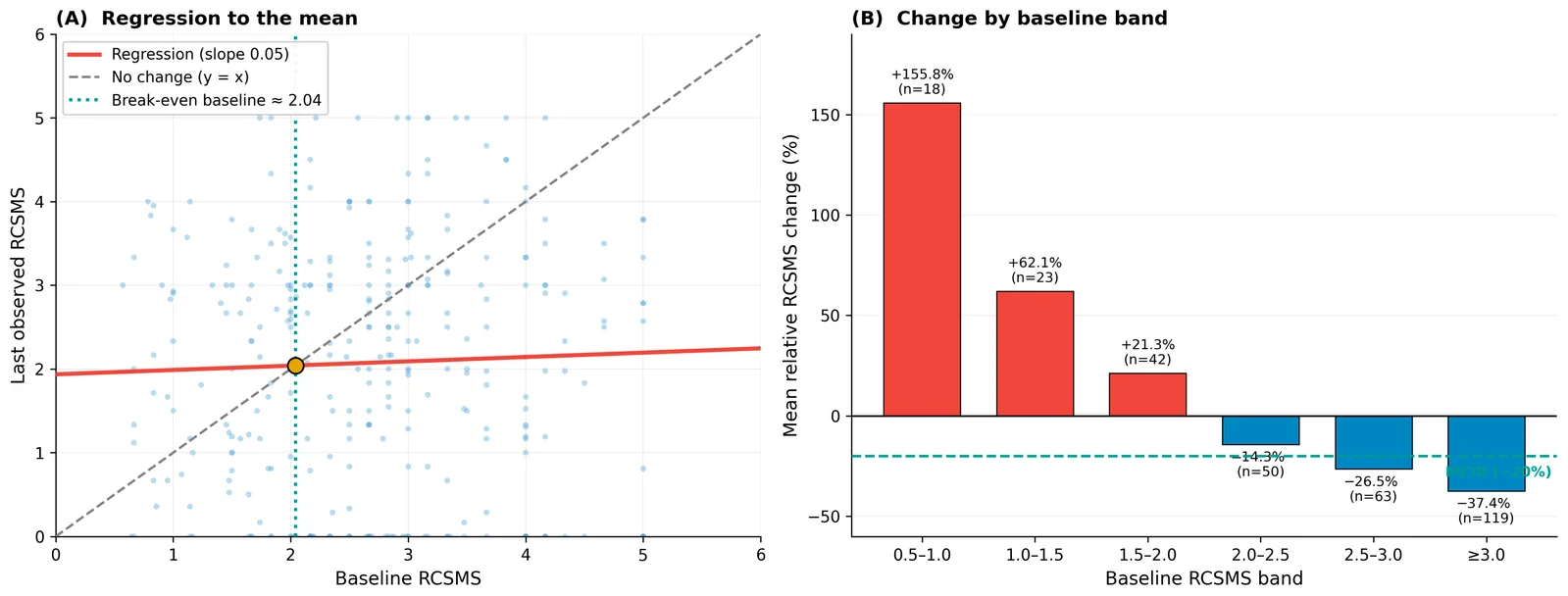
Regression to the mean in the RCSMS. (**A**) Last observed RCSMS versus baseline RCSMS for the 353 patients with at least one post-baseline observation: the follow-up regression line is nearly flat (slope 0.05), lying far below the identity (no-change) line (dashed), so high-baseline patients fall and low-baseline patients rise towards the cohort mean. The two lines cross at the break-even baseline (2.04, gold point; with a near-zero slope, this value approximates the cohort mean follow-up score); below it the RCSMS tends to worsen, and above it, to improve. (**B**) Mean relative RCSMS change by baseline band: the sign inverts at a baseline of 2, with an apparent worsening below the threshold and reductions beyond the −20% MCID above it (full gradient in Supplementary Table S4).

Regressing the last observed RCSMS on baseline RCSMS made the mechanism explicit (Figure 3A). The regression slope was 0.05, far below the value of 1 that would indicate a stable score, so patients starting high tended to fall and patients starting low tended to rise towards the cohort mean, the defining signature of regression to the mean. The follow-up line crossed the identity (no-change) line at a baseline of 2.04; below this value, the expected change in RCSMS is positive (apparent worsening), and above it, negative (improvement). The follow-up regression line crossed the identity line at an estimated baseline RCSMS of approximately 2.04. Because the regression slope was close to zero, this value primarily reflected the mean follow-up score of the cohort and should not be interpreted as a precisely estimated clinical cut-off. It was therefore considered compatible with, but not confirmatory of, the pre-specified threshold of 2.

The same inversion appears band by band (Figure 3B): mean relative change ran from +156% in the mildest patients to −37% in the most severe, crossing zero at a baseline of 2 and exceeding the −20% MCID only above it. In a categorical contrast computed on last observed values rather than on model-based month-24 estimates, patients with low baselines showed a mean change of +61.8% versus −29.4% in those with a baseline ≥ 2 (between-group difference highly significant). The break-even baseline derived from these data (2.04) was consistent with the independently pre-specified ≥2 threshold, while recognizing that this within-cohort analysis provides supportive rather than external validation.

### 3.5. Effect Sizes and Age Subgroups

**Figure 4:**
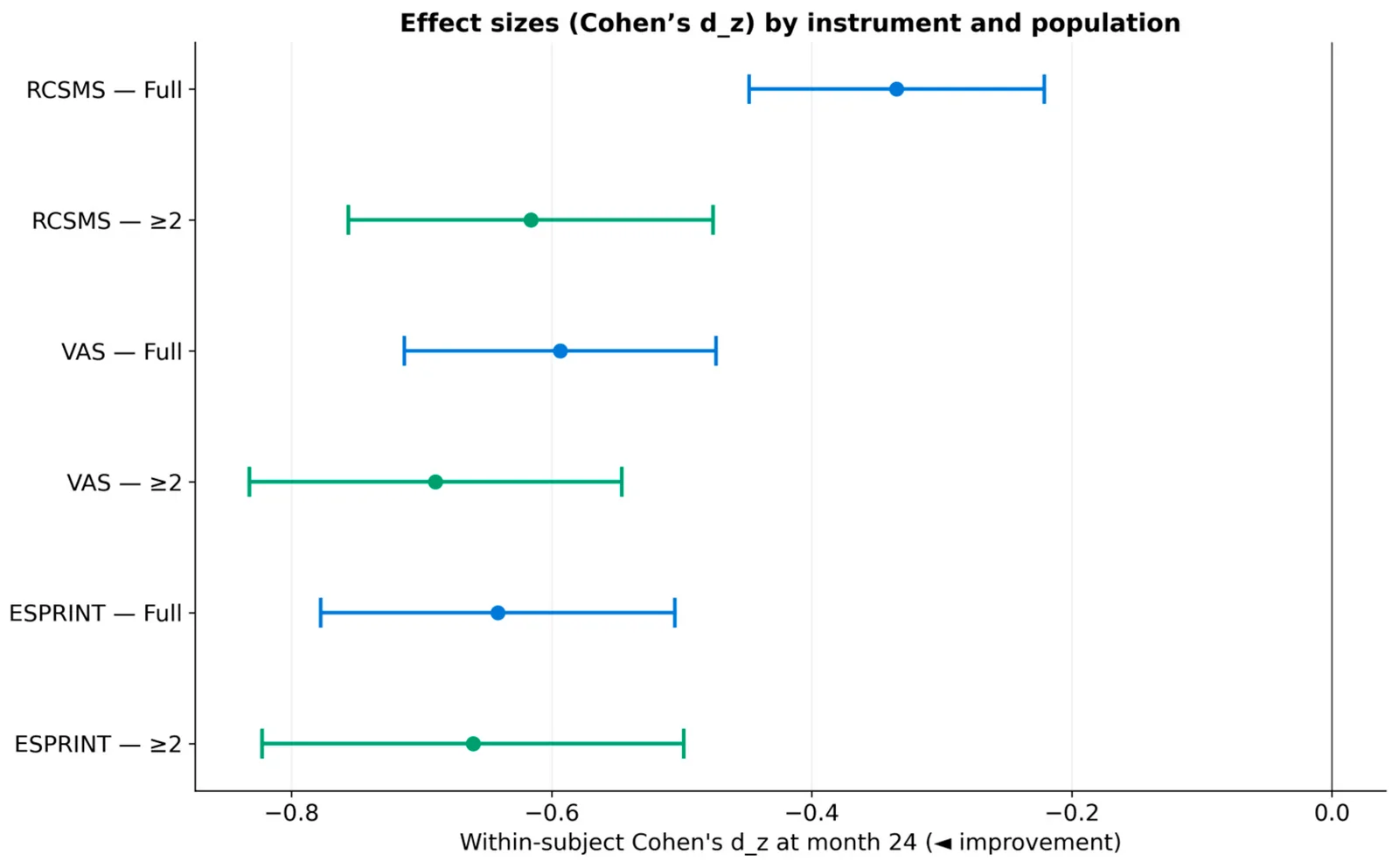
Forest plot of within-subject effect sizes (Cohen’s d_z with approximate 95% CI) at month 24, grouped by instrument, comparing the full cohort with the pre-specified ≥2 population. Restricting to ≥2 increases the effect size of all three instruments, most markedly for the bounded RCSMS (−0.33 [−0.44, −0.22] → −0.62 [−0.75, −0.49]), which crosses from below to beyond the medium (−0.5) threshold; the two populations overlap more for the unbounded instruments. Confidence intervals are approximated from the per-group sample size.

In the ≥2 population, within-subject effect sizes were medium for all three instruments (RCSMS d_z −0.62; ESPRINT-15 −0.66; VAS −0.69; Figure 4) and large when standardized by the baseline SD (Glass’s Δ). In the full cohort, the corresponding effect sizes were smaller, and the gap was greatest for the RCSMS (d_z −0.33 vs. −0.62), whereas the unbounded instruments differed little (ESPRINT-15 −0.59 vs. −0.66; VAS −0.55 vs. −0.69), indicating that the ≥2 restriction specifically restores the effect size of the RCSMS rather than generally inflating the response. Age-group analyses showed no significant age × time interaction in either population. Although one adjusted between-group contrast reached statistical significance, its magnitude was below the RCSMS MCID. These exploratory analyses are reported in Supplementary Table S5 and Figures S3 and S6.

### 3.6. Asthma

Among the 112 patients with concomitant asthma, the asthma composite score (ACSMS) decreased by approximately 48.8% at month 24 (MCID sustained from month 6), consistent with the rhinitis findings (Supplementary Figure S4; Supplementary Table S6).

An exploratory longitudinal analysis showed no evidence that the trajectory of the RCSMS differed between patients with and without concomitant asthma (Supplementary Table S10; MMRM asthma × visit interaction, *p* = 0.967).

## 4. Discussion

Clinically, in this real-world cohort, the polymerized triple-mite regimen was associated with substantial and concordant improvement in quality of life (ESPRINT-15) and global severity (VAS) across the entire cohort, including the low-baseline group. The RCSMS showed a clearer and earlier improvement in the pre-specified baseline ≥ 2 population. In patients with baseline RCSMS < 2, the discordance between the RCSMS and the other clinical measures, together with the component analysis, was consistent with reduced responsiveness of the composite score at low baseline values. These findings highlight the importance of baseline disease burden when interpreting combined symptom–medication scores in real-world AIT studies.

These findings have practical implications for the design and interpretation of real-world AIT studies. Because composite symptom–medication scores are bounded, effectiveness analyses including patients with very low baseline scores may underestimate measurable improvement and should therefore consider baseline disease burden.

### 4.1. The Real-World Design as Both Limitation and Strength

The retrospective, single-arm design is an important limitation, as it precludes causal inferences, and the observed changes cannot be solely attributed to treatment. Natural history, co-interventions, regression to the mean and unmeasured factors may have contributed. Conversely, this design in the real world allowed evaluation of the RCSMS across the broad severity range encountered in routine clinical practice, which was relevant to the methodological objective of this study [30].

These observations should be confirmed within a controlled trial that combines objective and patient-reported (subjective) criteria [13].

### 4.2. Generalisability

Generalisability is bounded by the allergen profile. The cohort comprises patients sensitized to and treated with this specific preparation against the three locally relevant mite species (*Dermatophagoides pteronyssinus*, *D. farinae* and *Blomia tropicalis*), and the findings should not be uncritically extrapolated to other allergens or to other preparations [3,9].

### 4.3. Strengths and Other Limitations

The strengths of this study include a large, unselected cohort; follow-up of up to 24 months; three complementary clinical outcomes; and sensitivity analyses. Among the remaining limitations, as the break-even analysis was derived from the same cohort, its agreement with the pre-specified ≥2 threshold should be regarded as supportive evidence rather than external validation. The retrospective single-extraction design resulted in progressively fewer observations at later visits; however, evaluated baseline characteristics were similar between patients with and without an available month-24 RCSMS assessment. Residual informative censoring cannot nevertheless be excluded. The age subgroup analyses were exploratory and should be interpreted cautiously, particularly given the smaller numbers at later visits.

The potential influence of additional aeroallergen sensitization, present in approximately one-third of the cohort, was likely limited by the exclusion of clinically relevant sensitization requiring additional allergen immunotherapy.

Finally, this report addressed effectiveness; tolerability, graded by standard systemic-reaction criteria [31], was not its focus and is reported separately. Adverse events were retrospectively identified from the clinical records. One mild local reaction was documented and resolved without treatment. As minor local reactions may not always be recorded in routine clinical practice, this finding should not be interpreted as an incidence estimate. The pre-specified sensitivity analyses are reported in Supplementary Table S7.

## 5. Conclusions

Polymerized subcutaneous immunotherapy with the three locally relevant mite species *(Dermatophagoides pteronyssinus*, *D. farinae* and *Blomia tropicalis*) was associated with clinically relevant improvement across complementary outcomes, being most clearly demonstrable on the RCSMS at baseline ≥ 2 and concordant on quality-of-life and global-severity measures. Baseline severity should, therefore, be considered when interpreting the bounded combined symptom–medication score in real-world allergen immunotherapy studies. The pre-specified baseline RCSMS ≥ 2 threshold was consistent within the cohort analyses, while external validation remains warranted. These findings should be confirmed through controlled trials.

## Figures and Tables

**Table 1:** Baseline characteristics by analysis population.

Characteristic	Full Cohort (*n* = 353)	RCSMS ≥ 2 (*n* = 259)	RCSMS < 2 (*n* = 94)
Female sex, *n* (%)	179 (50.7)	132 (51.0)	47 (50.0)
Age, years—mean (SD); median [IQR]	15.9 (12.3); 11 [8, 20]	15.2 (11.8); 10 [7, 18]	18.1 (13.8); 13 [9, 20.5]
Adults/adolescents/children, *n*	98/76/179	64/55/140	33/22/39
Additional aeroallergen sensitization *n* (%)	114 (32.3)	85 (32.8)	29 (30.9)
Concomitant asthma, *n* (%)	112 (31.7)	89 (34.4)	23 (24.5)
Atopic dermatitis, *n* (%)	48 (13.6)	35 (13.5)	13 (13.8)
Baseline RCSMS, mean (SD)	2.66 (1.05)	3.13 (0.80)	1.38 (0.40)
Baseline VAS, mean (SD)	6.03 (2.42)	6.23 (2.44)	5.48 (2.27)
Baseline ESPRINT-15, mean (SD)	2.60 (1.47) [*n* = 279]	2.79 (1.50) [*n* = 199]	2.12 (1.27) [*n* = 80]

**Table 2:** Month-24 MMRM results by instrument and population. Baseline and month-24 estimated marginal means (EMMs) with 95% confidence intervals, relative change (Δ%), within-subject Cohen’s d_z with its conventional interpretation, and the within-group change *p*-value (AR(1) covariance, REML). Estimates are associative (single-arm design).

Instrument	Population (*n*)	Baseline → M24 EMM [95% CI]	Δ%	Cohen’s d_z (Interpretation)	*p*
RCSMS	Full cohort (353)	2.657 → 1.719 [1.335, 2.122]	−35.3%	−0.33 (small)	<0.001
RCSMS	Baseline ≥ 2 (232)	3.129 → 1.618 [1.197, 2.039]	−48.3%	−0.62 (medium)	<0.001
RCSMS	Baseline < 2 (94)	—	↑ †	—	—
ESPRINT-15	Full cohort (279)	2.599 → 0.786 [0.364, 1.209]	−69.7%	−0.59 (medium)	<0.001
ESPRINT-15	Baseline ≥ 2 (178)	2.788 → 1.001 [0.410, 1.592]	−64.1%	−0.66 (medium)	<0.001
ESPRINT-15	Baseline < 2 (80)	—	↓ †	—	—
VAS	Full cohort (353)	5.992 → 2.959 [2.368, 3.550]	−50.6%	−0.55 (medium)	<0.001
VAS	Baseline ≥ 2 (232)	6.228 → 3.013 [2.210, 3.816]	−51.6%	−0.69 (medium)	<0.001
VAS	Baseline < 2 (94)	—	↓ †	—	—
ACSMS (asthma)	*n* = 112	1.442 → 0.738 [0.421, 1.056]	−48.8%	−0.53 (medium)	<0.001

EMM, estimated marginal mean (MMRM, AR(1), REML); Δ%, relative change from the baseline EMM. d_z is a within-subject Cohen’s d; the small/medium/large labels follow Cohen’s heuristic (≥0.2/≥0.5/≥0.8) applied to a within-subject effect size and are not between-group d; baseline-standardized (Glass’s Δ) effect sizes are large for every outcome, reflecting wide inter-individual variability. *p* tests change from baseline within each group; in this single-arm, uncontrolled design, it does not establish efficacy, and no effect size is attributable to treatment. † The baseline RCSMS < 2 group was excluded from the pre-specified primary analysis and is reported by direction only: the RCSMS rises (↑) while the VAS and ESPRINT-15 continue to improve (↓), a divergence consistent with a floor effect and regression to the mean rather than with a treatment effect. Comparisons across instruments, populations and timepoints are exploratory and unadjusted for multiplicity. MCID reached sustainably from month 12 (full-cohort RCSMS and VAS), month 3 (baseline ≥ 2 RCSMS and ESPRINT-15) and month 6 (ACSMS). Of the 259 patients with a baseline RCSMS ≥ 2, *n* = 232 had at least one post-baseline RCSMS observation and constitute the MMRM-analyzable set shown here.

## Data Availability

The data presented in this study are available on reasonable request from the corresponding author. The data are not publicly available because they consist of anonymized patient records collected under routine clinical care and are subject to the data-protection provisions of the ethics approval.

## Supplementary Materials

The following supporting information can be downloaded at: https://www.mdpi.com/article/10.3390/jcm15166383/s1. Supplementary Tables: Table S1. RCSMS by visit and population. Table S2. ESPRINT-15 by visit and population. Table S3. VAS by visit and population. Table S4. Regression-to-the-mean gradient. Table S5. Age subgroups. Table S6. Asthma composite score (ACSMS). Table S7. Sensitivity analyses. Table S8. Additional aeroallergen sensitisation by analysis population. Table S9. Observed RCSMS symptom and medication components by baseline population. Table S10. Observed RCSMS by asthma status. Table S11. Baseline characteristics according to availability of a month-24 RCSMS assessment. Table S12. Month-24 MMRM summary (all instruments and populations). Supplementary Figures: Figure S1. ESPRINT-15 trajectory (mean ± 95% CI) by population. Figure S2. VAS trajectory (mean ± 95% CI) by population. Figure S3. RCSMS trajectory by age group in the full cohort. Trajectory shape did not differ significantly by age (age × time interaction, likelihood-ratio *p* = 0.407). Figure S4. ACSMS trajectory among asthmatics (*n* = 112). Figure S5. Number of patients with an observed RCSMS at each visit, illustrating administrative right-censoring from staggered entry and a single data extraction (not dropout). Figure S6. Month-24 RCSMS change by age group in the full cohort versus the ≥2 population.

## Abbreviations

The following abbreviations are used in this manuscript:

ACSMS	Asthma Combined Symptom–Medication Score
AIT	Allergen Immunotherapy
AR	Allergic Rhinoconjunctivitis
HDM	House Dust Mite
MCID	Minimal Clinically Important Difference
RCSMS	Rhinoconjunctivitis Combined Symptom–Medication Score
RMS	Rhinoconjunctivitis Medication Score
RSS	Rhinoconjunctivitis Symptom Score
SCIT	Subcutaneous Immunotherapy
TUs	Therapeutic Units
VAS	Visual Analogue Scale
