# Supplementary material for: Real-World Effectiveness of Polymerized House-Dust-Mite Subcutaneous Immunotherapy up to 24 Months: Why Baseline Symptom Severity Shapes Measurement of the Combined Symptom–Medication Response
Source: J Clin Med. 2026 Aug 18;15(16):6383. doi: 10.3390/jcm15166383 (PMC13514145; doi:10.3390/jcm15166383)
Supplement: Supplementary file 1 [file jcm-15-06383-s001.zip › jcm-4479627-supplementary.pdf]

Supplementary Material

Real-world effectiveness of polymerised house-dust-mite subcutaneous immunotherapy up to 24 months: why baseline symptom severity shapes measurement of the combined symptom–medication response

Supplementary Tables 2

Table S1. RCSMS by visit and population 2

Table S2. ESPRINT-15 by visit and population 2

Table S3. VAS by visit and population 2

Table S4. Regression-to-the-mean gradient 3

Table S5. Age subgroups 3

Table S6. Asthma composite score (ACSMS) 3

Table S7. Sensitivity analyses 3

Table S8. Additional aeroallergen sensitisation by analysis population. 4

Table S9. Observed RCSMS symptom and medication components by baseline population 4

Table S10. Observed RCSMS by asthma status 4

Table S11. Baseline characteristics according to availability of a month-24 RCSMS assessment. 5

Table S12. Month-24 MMRM summary (all instruments and populations) 5

Supplementary Figures 6

Figure S1. ESPRINT-15 trajectory (mean ±95% CI) by population. 6

Figure S2. VAS trajectory (mean ±95% CI) by population. 6

Figure S3. RCSMS trajectory by age group in the full cohort. Trajectory shape did not differ significantly by age (age × time interaction, likelihood-ratio p=0.407). 7

Figure S4. ACSMS trajectory among asthmatics (n=112). 7

Figure S5. Number of patients with an observed RCSMS at each visit, illustrating administrative right-censoring from staggered entry and a single data extraction (not dropout). 8

Figure S6. Month-24 RCSMS change by age group in the full cohort versus the ⩾2 population 8

Supplementary Tables

Table S1. RCSMS by visit and population

RCSMS observed mean (SD) [n] at scheduled visits. M0 = baseline.

Month	Full cohort	RCSMS ≥2	RCSMS <2
M0	2.66 (1.05) [n=353]	3.13 (0.80) [n=259]	1.38 (0.40) [n=94]
M2	2.36 (1.09) [n=315]	2.56 (1.04) [n=232]	1.81 (1.06) [n=83]
M3	2.12 (1.17) [n=281]	2.29 (1.16) [n=208]	1.63 (1.07) [n=73]
M6	2.14 (1.16) [n=208]	2.22 (1.21) [n=158]	1.89 (0.95) [n=50]
M12	1.94 (1.34) [n=139]	1.88 (1.33) [n=106]	2.14 (1.38) [n=33]
M18	2.10 (1.54) [n=97]	2.22 (1.56) [n=75]	1.69 (1.42) [n=22]
M24	1.71 (1.62) [n=67]	1.60 (1.58) [n=53]	2.13 (1.77) [n=14]

Table S2. ESPRINT-15 by visit and population

ESPRINT-15 observed mean (SD) [n].

Month	Full cohort	RCSMS ≥2	RCSMS <2
M0	2.60 (1.47) [n=279]	2.79 (1.50) [n=199]	2.12 (1.27) [n=80]
M2	1.83 (1.35) [n=250]	1.92 (1.35) [n=178]	1.59 (1.33) [n=72]
M3	1.69 (1.38) [n=224]	1.80 (1.36) [n=161]	1.40 (1.39) [n=63]
M6	1.50 (1.22) [n=169]	1.56 (1.32) [n=124]	1.33 (0.92) [n=45]
M12	1.27 (1.18) [n=100]	1.26 (1.21) [n=74]	1.27 (1.10) [n=26]
M18	1.27 (1.35) [n=54]	1.23 (1.27) [n=40]	1.37 (1.58) [n=14]
M24	0.72 (1.07) [n=26]	0.90 (1.24) [n=15]	0.49 (0.78) [n=11]

Table S3. VAS by visit and population

VAS observed mean (SD) [n].

Month	Full cohort	RCSMS ≥2	RCSMS <2
M0	6.03 (2.42) [n=353]	6.23 (2.44) [n=259]	5.48 (2.27) [n=94]
M2	5.00 (2.35) [n=314]	5.02 (2.37) [n=231]	4.92 (2.28) [n=83]
M3	4.59 (2.51) [n=275]	4.63 (2.51) [n=205]	4.44 (2.55) [n=70]
M6	4.66 (2.42) [n=208]	4.47 (2.43) [n=158]	5.26 (2.31) [n=50]
M12	3.61 (2.50) [n=135]	3.50 (2.56) [n=102]	3.96 (2.30) [n=33]
M18	3.76 (2.74) [n=95]	3.85 (2.66) [n=74]	3.43 (3.05) [n=21]
M24	2.95 (2.88) [n=66]	2.97 (3.02) [n=52]	2.91 (2.37) [n=14]

Table S4. Regression-to-the-mean gradient

Mean relative change in RCSMS (last available visit vs baseline) by baseline band, illustrating regression to the mean. The sign reverses between the 1.5–2 and 2–2.5 bands; estimated break-even baseline ≈ 2.04.

Baseline band	n	Mean Δ% (last visit)
0.5–1	18	+155.8%
1–1.5	23	+62.1%
1.5–2	42	+21.3%
2–2.5	50	-14.3%
2.5–3	63	-26.5%
≥3	119	-37.4%

Table S5. Age subgroups

Observed relative RCSMS changes at months 12 and 24 by age group (corrected age classification). Values are descriptive and based on the observations available at each visit. The age-group × time interaction was not statistically significant in either population (likelihood-ratio test, models fitted by maximum likelihood: full cohort p=0.407; baseline RCSMS ≥2 p=0.695). Adjusted contrasts using all post-baseline observations were: adolescents vs adults β=−0.303, p=0.013 (Bonferroni p=0.038); children vs adults β=−0.090, p=0.380; adolescents vs children β=−0.213, p=0.044 (Bonferroni p=0.132). All three age groups exceeded the RCSMS MCID in the baseline RCSMS ≥2 population. Month-24 values are descriptive and should be interpreted cautiously because of the smaller sample sizes. Exploratory and post-hoc.

Age group	Full cohort Δ% at month 12	Full cohort Δ% at month 24	Baseline RCSMS ≥2 Δ% at month 12	Baseline RCSMS ≥2 Δ% at month 24
Adults	-27.6% (n = 34)	-39.7% (n = 19)	-48.4% (n = 22)	-47.8% (n = 13)
Adolescents	-39.4% (n = 33)	-32.9% (n = 12)	49.8% (n = 25)	-57.1% (n = 10)
Children	-21.5% (n = 72)	-34.8% (n = 36)	-32.2% (n = 59)	-46.5% (n = 30)

Table S6. Asthma composite score (ACSMS)

ACSMS observed mean (SD) [n] among asthmatics (n=112). MMRM-adjusted month-24 change −48.8%; MCID sustained from month 6.

Month	n	ACSMS mean (SD)
M0	112	1.46 (0.96)
M2	96	1.37 (0.97)
M3	84	1.27 (0.90)
M6	62	1.17 (0.99)
M12	46	0.81 (0.78)
M18	35	0.73 (0.68)
M24	22	0.72 (0.80)

Table S7. Sensitivity analyses

Robustness of the primary ≥2 estimate across MMRM,[25,26] complete-case, last-observation/LOCF and tipping-point/delta-MI analyses. No baseline-observation-carried-forward (BOCF) analysis was performed: under administrative right-censoring, reversion to baseline is not a plausible assumption and would bias towards the null.

Sensitivity analysis	RCSMS ≥2 (M24)	VAS ≥2 (M24)	ESPRINT-15 ≥2 (M24)
**Primary MMRM (AR(1), REML)**	−1.511 (−48.3%)*	−3.215 (−51.6%)*	−1.787 (−64.1%)*
**Complete-case at endpoint (≥12 m)**	−1.261 (1.76); n=106	−3.009 (3.86); n=105	−1.648 (1.79); n=74

Sensitivity analysis	RCSMS ≥2 (M24)	VAS ≥2 (M24)	ESPRINT-15 ≥2 (M24)
**Last-observation / LOCF**	−0.926 (1.62); n=259	−2.130 (3.34); n=259	−1.060 (1.74); n=199
**Tipping-point / delta-MI (MAR stress test)**	Robust (MCID lost only if unobserved ≈0.5 pts worse)	—	—

* p < 0.001. MMRM shows the estimated marginal-mean change (relative change); complete-case and last-observation/LOCF show the observed mean change (SD) with the number of patients (n) per scenario; LOCF is numerically equivalent to the last-observation analysis. All estimates refer to the pre-specified baseline RCSMS ≥2 population.

Table S8. Additional aeroallergen sensitisation by analysis population.

Additional aeroallergen sensitisation	Full cohort (n = 353)	Baseline RCSMS ≥2 (n = 259)	Baseline RCSMS <2 (n = 94)
**Any additional aeroallergen sensitisation, n (%)**	114 (32.3)	85 (32.8)	29 (30.9)
**Animal epithelia, n (%)**	91 (25.8)	71 (27.4)	20 (21.3)
**Cockroach, n (%)**	33 (9.3)	23 (8.9)	10 (10.6)
**Grass pollen, n (%)**	2 (0.6)	2 (0.8)	0 (0.0)

Animal epithelia included sensitisation recorded as animal epithelia, cat or dog in the clinical database. Patients could have more than one additional aeroallergen sensitisation; therefore, categories are not mutually exclusive and percentages do not sum to the total percentage.

Table S9. Observed RCSMS symptom and medication components by baseline population

Population and component	Baseline	Month 3	Month 6	Month 12	Month 24	Baseline–month 24 difference*
**Baseline RCSMS ≥2**						
Patients observed, n	259	208	158	106	53	53
**RSS, mean (SD)**	1.531 (0.753)	1.038 (0.745)	1.051 (0.751)	0.865 (0.772)	0.770 (0.912)	−0.679
**RMS, mean (SD)**	1.598 (0.558)	1.251 (0.773)	1.171 (0.769)	1.016 (0.845)	0.827 (0.871)	−0.788
**RCSMS, mean (SD)**	3.129 (0.802)	2.290 (1.162)	2.222 (1.214)	1.881 (1.332)	1.598 (1.581)	−1.468
**Baseline RCSMS <2**						
Patients observed, n	94	73	50	33	14	14
**RSS, mean (SD)**	0.920 (0.457)	0.863 (0.716)	1.017 (0.605)	1.111 (0.790)	0.881 (0.966)	−0.012
**RMS, mean (SD)**	0.461 (0.484)	0.766 (0.696)	0.873 (0.701)	1.030 (0.842)	1.250 (0.920)	+0.944
**RCSMS, mean (SD)**	1.381 (0.404)	1.629 (1.069)	1.890 (0.949)	2.141 (1.382)	2.131 (1.772)	+0.932

**Paired baseline–month 24 differences were calculated among patients with observations at both time points (n=53 for baseline RCSMS ≥2; n=14 for baseline RCSMS <2). RSS, Rhinoconjunctivitis Symptom Score; RMS, Rescue Medication Score; RCSMS, Rhinoconjunctivitis Combined Symptom–Medication Score. Values at each visit are observed means (SD).*

Table S10. Observed RCSMS by asthma status

Visit	Without asthma (n = 241)	With asthma (n = 112)
**Baseline**	2.638 (1.058) [n = 241]	2.720 (1.050) [n = 112]
**Month 3**	2.081 (1.179) [n = 197]	2.206 (1.162) [n = 84]
**Month 6**	2.125 (1.148) [n = 146]	2.183 (1.204) [n = 62]
**Month 12**	1.853 (1.302) [n = 93]	2.125 (1.421) [n = 46]
**Month 24**	1.659 (1.585) [n = 45]	1.813 (1.734) [n = 22]
Longitudinal group × time interaction MMRM (AR(1), REML) p = 0.967		

Values are observed mean (SD) [n]. The difference in RCSMS trajectories according to concomitant asthma was evaluated using a mixed model

for repeated measures (MMRM) with an AR(1) covariance structure estimated by REML. The model included visit, asthma status, the asthma × visit interaction, baseline RCSMS and age group as fixed effects. The non-significant interaction indicates no evidence that the trajectory differed according to asthma status.

Table S11. Baseline characteristics according to availability of a month-24 RCSMS assessment.

Baseline characteristic	Month-24 RCSMS available (n = 67)	No month-24 RCSMS (n = 286)	p value
**Female sex, n (%)**	38 (56.7)	141 (49.3)	0.339
**Adults, n (%)**	19 (28.4)	79 (27.6)	
**Adolescents, n (%)**	12 (17.9)	64 (22.4)	0.718*
**Children, n (%)**	36 (53.7)	143 (50.0)	
**Baseline RCSMS, mean (SD)**	2.68 (1.06)	2.66 (1.06)	0.832
**Baseline VAS, mean (SD)**	5.99 (2.17)	6.04 (2.48)	0.993

*Values are n (%) or mean (SD). p values were obtained using the chi-square test for categorical variables and the Mann-Whitney U test for continuous variables. *Overall comparison of age-group distribution.*

Table S12. Month-24 MMRM summary (all instruments and populations)

Month-24 MMRM estimated marginal means (EMM) with 95% confidence intervals, relative change (Δ%), within-subject Cohen's d_z with its conventional interpretation, and the within-group change p-value (AR(1), REML). Estimates are associative (single-arm design); p tests change from baseline and does not establish efficacy. Labels are Cohen's heuristic applied to a within-subject effect size (not between-group d); Glass's Δ is large for every outcome. † RCSMS in the <2 group rises a pattern consistent with floor and regression-to-the-mean effects.. ‡ ESPRINT-15 and VAS changes in the <2 group are descriptive. Of the 259 patients with a baseline RCSMS ≥2, n = 232 had at least one post-baseline RCSMS observation and constitute the MMRM-analysable set shown here. Mirrors Table 2 of the main manuscript.

Instrument	Population (n)	Baseline → M24 EMM [95% CI]	Δ%	Cohen's d_z (interpretation)	p
**RCSMS**	Full cohort (353)	2.657 → 1.719 [1.335, 2.122]	−35.3%	−0.33 (small)	<0.001
**RCSMS**	Baseline ≥2 (232)	3.129 → 1.618 [1.197, 2.039]	−48.3%	−0.62 (medium)	<0.001
**RCSMS**	Baseline <2 (94)	—	+64.2%	not interpretable †	—
**ESPRINT-15**	Full cohort (279)	2.599 → 0.786 [0.364, 1.209]	−69.7%	−0.59 (medium)	<0.001
**ESPRINT-15**	Baseline ≥2 (178)	2.788 → 1.001 [0.410, 1.592]	−64.1%	−0.66 (medium)	<0.001
**ESPRINT-15**	Baseline <2 (80)	—	−83.2%	descriptive ‡	—
**VAS**	Full cohort (353)	5.992 → 2.959 [2.368, 3.550]	−50.6%	−0.55 (medium)	<0.001
**VAS**	Baseline ≥2 (232)	6.228 → 3.013 [2.210, 3.816]	−51.6%	−0.69 (medium)	<0.001
**VAS**	Baseline <2 (94)	—	−45.7%	descriptive ‡	—
**ACSMS (asthma)**	n=112	1.442 → 0.738 [0.421, 1.056]	−48.8%	−0.53 (medium)	<0.001

Supplementary Figures

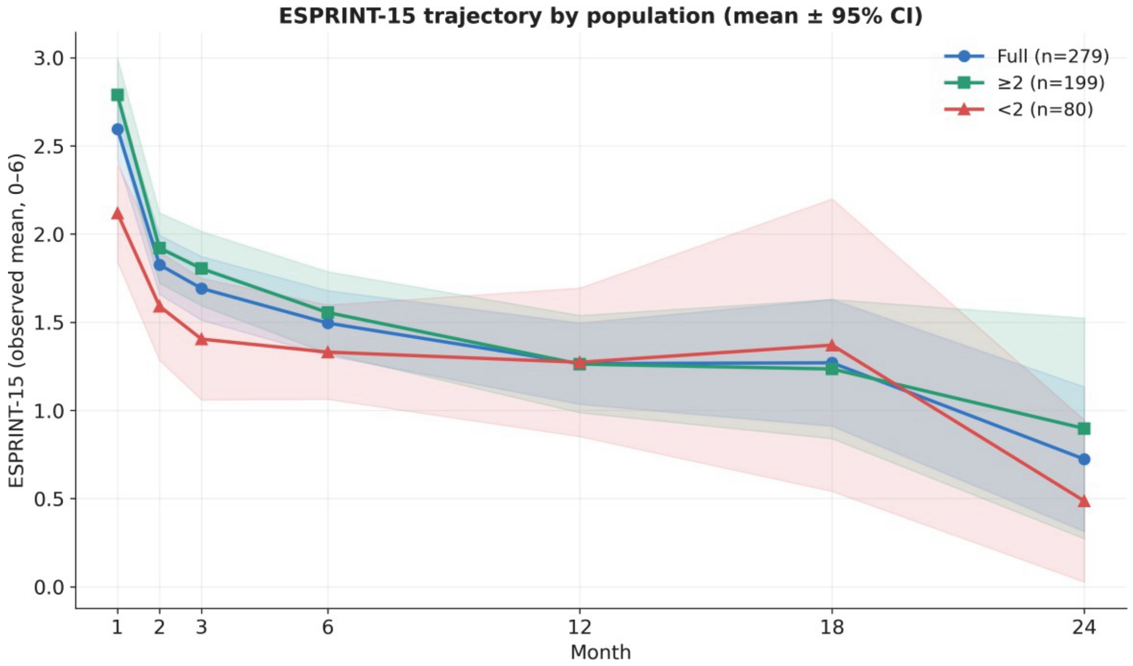

Figure S1. ESPRINT-15 trajectory (mean ±95% CI) by population.

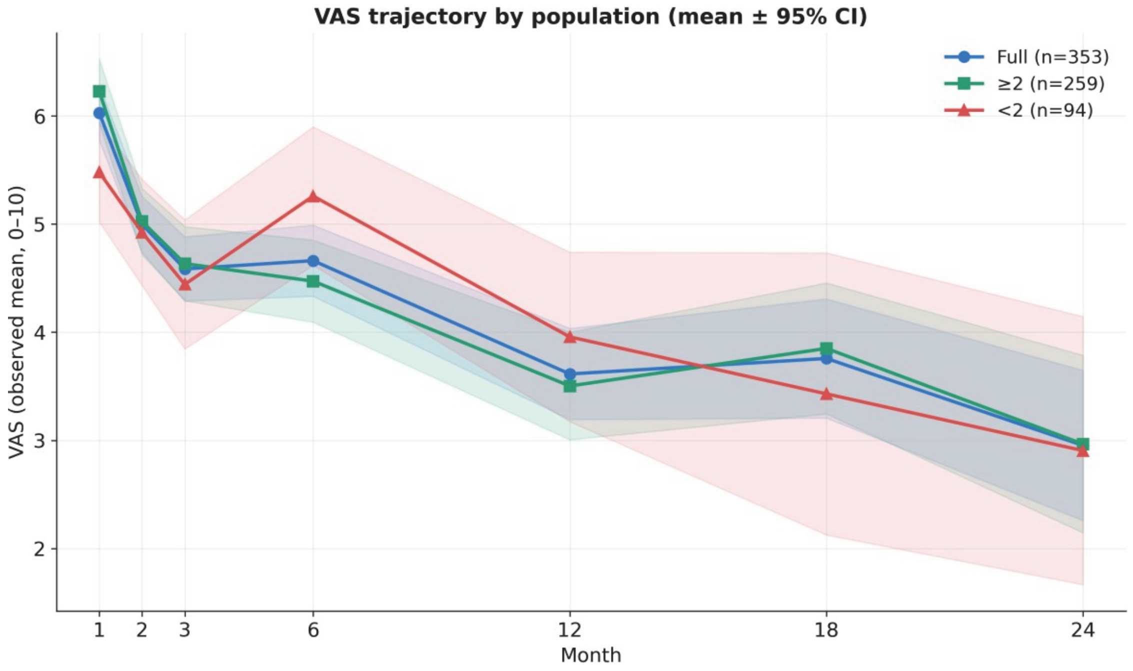

Figure S2. VAS trajectory (mean ±95% CI) by population.

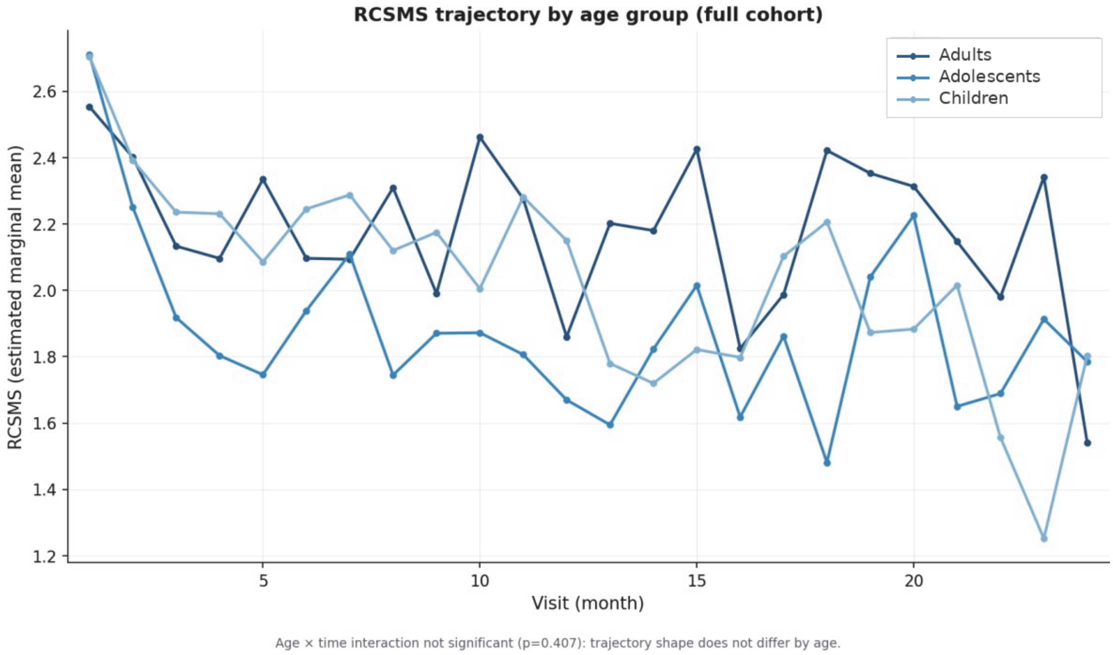

Figure S3. RCSMS trajectory by age group in the full cohort. Trajectory shape did not differ significantly by age (age × time interaction, likelihood-ratio p=0.407).

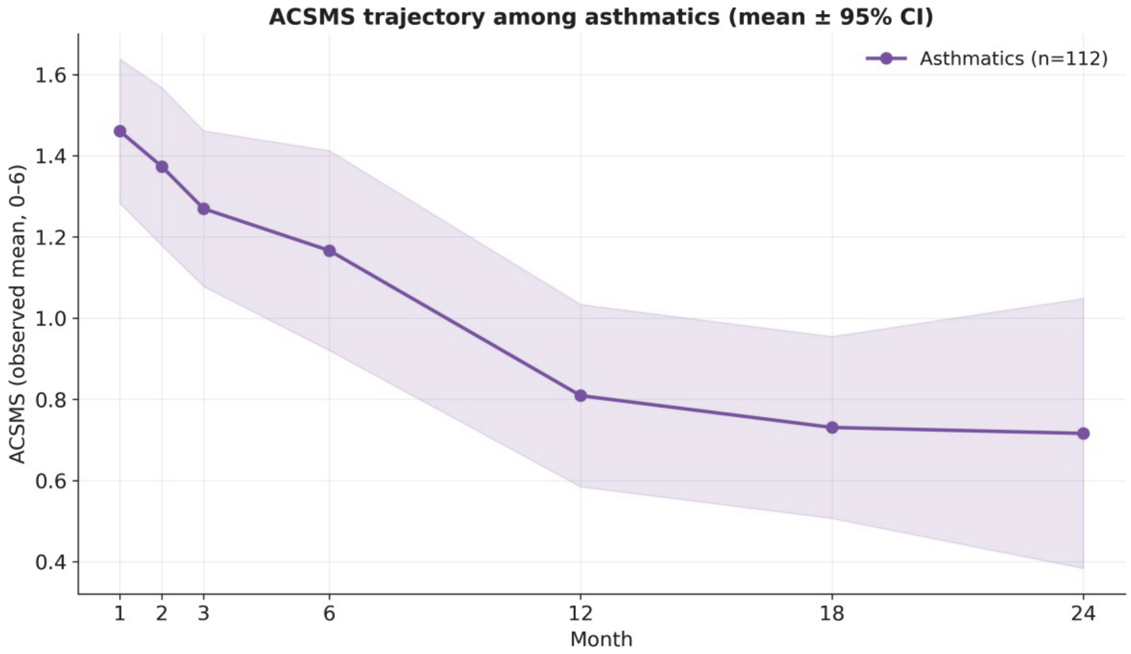

Figure S4. ACSMS trajectory among asthmatics (n=112).

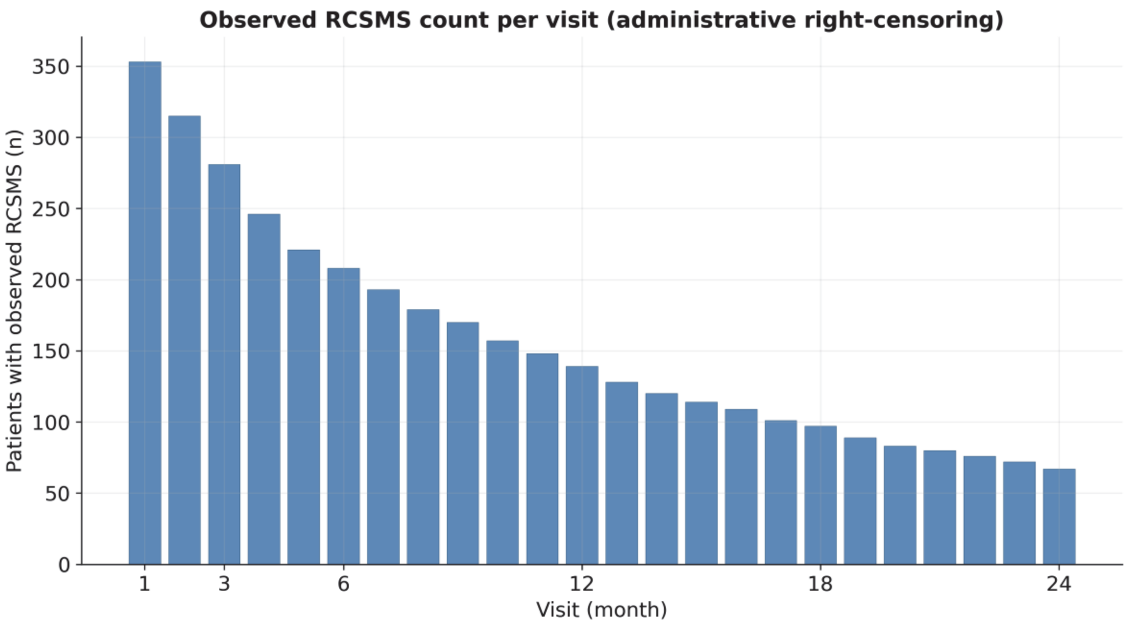

Figure S5. Number of patients with an observed RCSMS at each visit, illustrating administrative right-censoring from staggered entry and a single data extraction (not dropout).

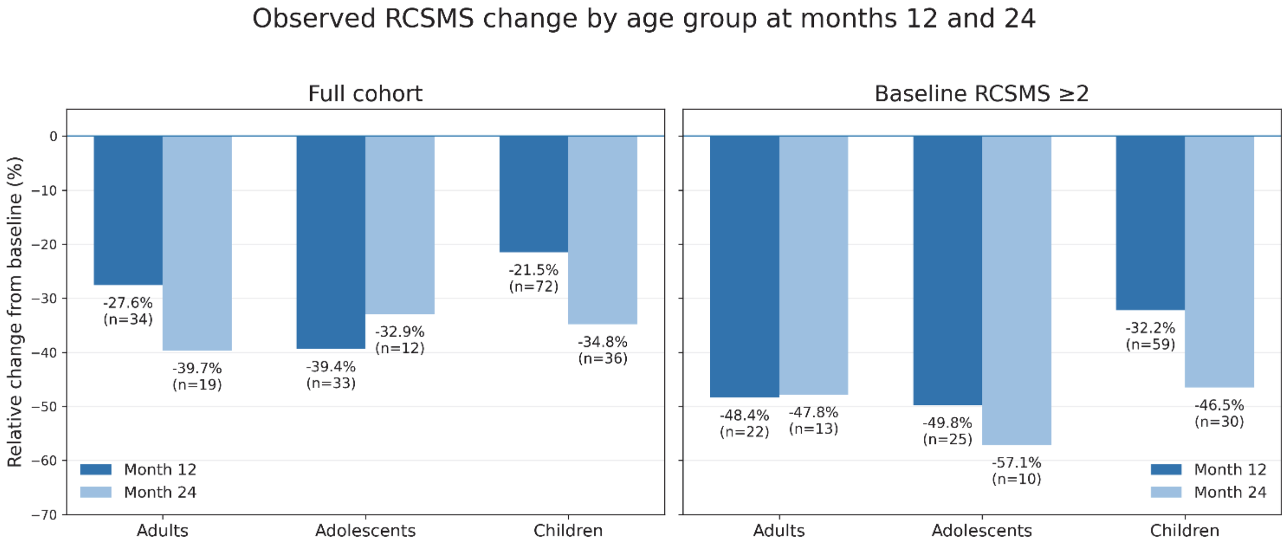

Figure S6. RCSMS change at months 12 and 24 by age group in the full cohort versus the ≥2 population
